# Stem rust on barberry species in Europe: Host specificities and genetic diversity

**DOI:** 10.3389/fgene.2022.988031

**Published:** 2022-09-27

**Authors:** Julian Rodriguez-Algaba, Mogens S. Hovmøller, Philipp Schulz, Jens G. Hansen, Juan Antonio Lezáun, Jessica Joaquim, Biagio Randazzo, Paweł Czembor, Liga Zemeca, Svetlana Slikova, Alena Hanzalová, Sarah Holdgate, Sarah Wilderspin, Fabio Mascher, Frederic Suffert, Marc Leconte, Kerstin Flath, Annemarie F. Justesen

**Affiliations:** ^1^ Department of Agroecology, Faculty of Science and Technology, Aarhus University, Slagelse, Denmark; ^2^ Federal Research Centre for Cultivated Plants, Julius Kühn-Institut, Institute for Plant Protection in Field Crops and Grassland, Kleinmachnow, Germany; ^3^ INTIA, Institute for Agrifood Technology and Infrastructures of Navarra, Villava, Navarra, Spain; ^4^ Agroscope, Crop Plant Breeding and Genetic Ressources, Nyon, Switzerland; ^5^ Società Semplice Agricola Randazzo, Baucina, Italy; ^6^ Plant Breeding and Acclimatization Institute-National Research Institute, Radzików, Poland; ^7^ Institute of Plant Protection Research “Agrihorts”, Latvia University of Life Sciences and Technologies, Jelgava, Latvia; ^8^ National Agricultural and Food Centre, Bratislava, Slovakia; ^9^ Crop Research Institute, Department of Genetics and Plant Breeding Methods, Prague, Czech Republic; ^10^ National Institute of Agricultural Botany (NIAB), Cambridge, United Kingdom; ^11^ INRAE (French National Institute for Agriculture Food and Environment), Université Paris-Saclay, Thiverval-Grignon, France

**Keywords:** *Puccinia graminis*, alternate host, sexual recombination, berberis, elongation factor (EF1-α) gene

## Abstract

The increased emergence of cereal stem rust in southern and western Europe, caused by the pathogen *Puccinia graminis*, and the prevalence of alternate (sexual) host, *Berberis* species, have regained attention as the sexual host may serve as source of novel pathogen variability that may pose a threat to cereal supply. The main objective of the present study was to investigate the functional role of *Berberis* species in the current epidemiological situation of cereal stem rust in Europe. Surveys in 11 European countries were carried out from 2018 to 2020, where aecial infections from five barberry species were collected. Phylogenetic analysis of 121 single aecial clusters of diverse origin using the elongation factor 1-α gene indicated the presence of different special forms (aka *formae speciales*) of *P. graminis* adapted to different cereal and grass species. Inoculation studies using aecial clusters from Spain, United Kingdom, and Switzerland resulted in 533 stem rust isolates sampled from wheat, barley, rye, and oat, which confirmed the presence of multiple special forms of *P. graminis*. Microsatellite marker analysis of a subset of 192 sexually-derived isolates recovered on wheat, barley and rye from the three populations confirmed the generation of novel genetic diversity revealed by the detection of 135 multilocus genotypes. Discriminant analysis of principal components resulted in four genetic clusters, which grouped at both local and country level. Here, we demonstrated that a variety of *Berberis* species may serve as functional alternate hosts for cereal stem rust fungi and highlights the increased risks that the sexual cycle may pose to cereal production in Europe, which calls for new initiatives within rust surveillance, epidemiological research and resistance breeding.

## Introduction

Stem rust, caused by the basidiomycete *Puccinia graminis,* is a major threat to cereal production globally (Singh et al., 2016; Saunders et al., 2019; Patpour et al., 2022). Stem rust of cereals present unique features in its life cycle including asexual reproduction on the primary cereal host and sexual reproduction on a phylogenetically unrelated alternate host (Stubbs et al., 1985; Jin et al., 2010). Sexual reproduction provides advantages regarding the generation of novel genetic and virulence combinations that may endanger the durability of cereal rust resistance and a stable cereal supply (Burdon and Roelfs, 1985; Billiard et al., 2012; Zhao et al., 2016). Additionally, the presence of the alternate host increases the level of initial fungal inoculum and advances the onset of the disease (Roelfs, 1982; Peterson et al., 2005). To date, approximately 100 species belonging to the *Berberidaceae* family, specifically to the *Berberis* and *Mahonia* genera, and interspecific hybrids, have been confirmed susceptible to *P. graminis* (Ahrendt, 1961; Roelfs et al., 1985; Zhao et al., 2016). This includes *Berberis vulgaris* (European or common barberry) that is the most widely distributed barberry species in Europe. The incidence of common barberry in Europe has significantly increased due to the repeal of eradication campaigns and legislation banning the planting of barberry plants near cereal crops and its re-introduction promoted by natural conservationists (Berlin et al., 2013b; Saunders et al., 2019). Besides common barberry, indigenous barberry species such as *B. vulgaris* subsp*. seroi* and *B. vulgaris* subsp*. australis* in Spain (López González, 1986) and *B. aetnensis* in Italy (Ahrendt, 1961), and interspecific hybrids, are present in Europe and may potentially play a role in the epidemiology of cereal rust fungi (Villegas et al., 2022).

The implementation of barberry eradication programs during the 20th century resulted in a reduction in the initial inoculum and the genetic variability generated in the alternate host, and ultimately in the stabilization of *P. graminis* races (Stakman, 1923; Hermansen, 1968; Roelfs, 1982). Consequently, no significant outbreaks of cereal stem rust have been reported in recent decades and the pathogen has been considered as a defeated enemy due to the combined effect of barberry eradication and the deployment of stem rust resistant varieties (Stakman, 1923; Peterson et al., 2005; Peterson, 2018). Unfortunately, the emergence and posterior evolution and spread of a highly virulent race in East Africa in 1998, commonly known as Ug99, left 90% of the global wheat germplasm susceptible to stem rust (Pretorius et al., 2000; Singh et al., 2011). Recently, stem rust has resurged in Western Europe causing multiple outbreaks on particularly bread and durum wheat and it is now considered as a re-emerged cereal pathogen (Patpour et al., 2022), e.g., Italy (Bhattacharya, 2017), Sweden (Berlin et al., unpublished data), and Germany (Olivera Firpo et al., 2017). Additionally, the role of the alternate host on cereal stem rust variability has regained attention due to the detection of pathogen races carrying unusual virulence combinations associated with the presence of the alternate host in Europe and beyond (Berlin et al., 2013b; Berlin et al., 2014; Olivera Firpo et al., 2017; Olivera et al., 2019; Patpour et al., 2022).

*P. graminis* comprises special forms (aka *formae speciales*, ff.spp.) adapted to certain cereal hosts, such as *P. graminis* f.sp. *tritici* infecting wheat (*Pgt*), *P. graminis* f.sp. *secalis* infecting rye (*Pgs*) and *P. graminis* f.sp. *avenae* infecting oat (*Pga*) (Eriksson and Henning, 1894; Anikster, 1984). *Berberis* species are alternate hosts for these ff.spp. in addition to several other *Puccinia* species infecting multiple cereals and grasses, e.g., *P. striiformis, P. brachypodii,* and *P. arrhenatheri* (Cummins and Greene, 1966; Naef et al., 2002; Jin et al., 2010; Rodriguez-Algaba et al., 2021). However, the genetic subdivision of these *Puccinia* species is still poorly understood (Berlin et al., 2013a). Since the aecial structures formed on barberry species often have a similar morphology, molecular-based methods such as DNA fingerprinting in addition to *in vivo* experiments using aecial samples *via* inoculation on cereal and grass hosts could be used to identify the cereal rust species that undergo sexual reproduction (Berlin et al., 2017; Lewis et al., 2018). Additionally, by applying molecular approaches such as neutral molecular markers on sexually-derived cereal rust samples, the genetic variability generated among fungal samples and populations can be determined (Zambino et al., 2000; Rodriguez-Algaba et al., 2017). DNA sequencing using both the internal transcribed spacer (ITS) region and the translation elongation factor 1-α (EF1-α) gene have previously been used to identify ff.spp. of *P. graminis* formed in the cereal/grass host (Zambino and Szabo, 1993; Abbasi et al., 2005). However, less attention has been put to characterize the aecial structures formed on barberry species and infer in the rust species that may undergo sexual reproduction. To date, only a few studies have reported on the *Puccinia* species and/or genera infecting barberry species in local areas in Europe (Berlin et al., 2013a; Lewis et al., 2018; Villegas et al., 2022).

The increased emergence of stem rust coupled with renewed prevalence of the alternate host in cereal growing areas in Europe have recently drawn the attention due to the detrimental role that the alternate host may pose to cereal supply. The main objective of the present study was to investigate the potential role of *Berberis* species in the epidemiology of cereal rust fungi by identifying the rust genera and/or species that infect the alternate host across Europe. Additionally, we evaluated which rust species may infect major cereal crops after sexual reproduction on *Berberis* spp., and investigated the genetic diversity generated on the cereal host. This fundamental knowledge on the role of the alternate host in cereal rust epidemiology will be crucial as sexual reproduction potentially increase genetic variability in cereal rust populations, and thereby increase the risks of break-down of rust resistance in crop varieties.

## Materials and methods

### Sample and data collection

**FIGURE 1:**
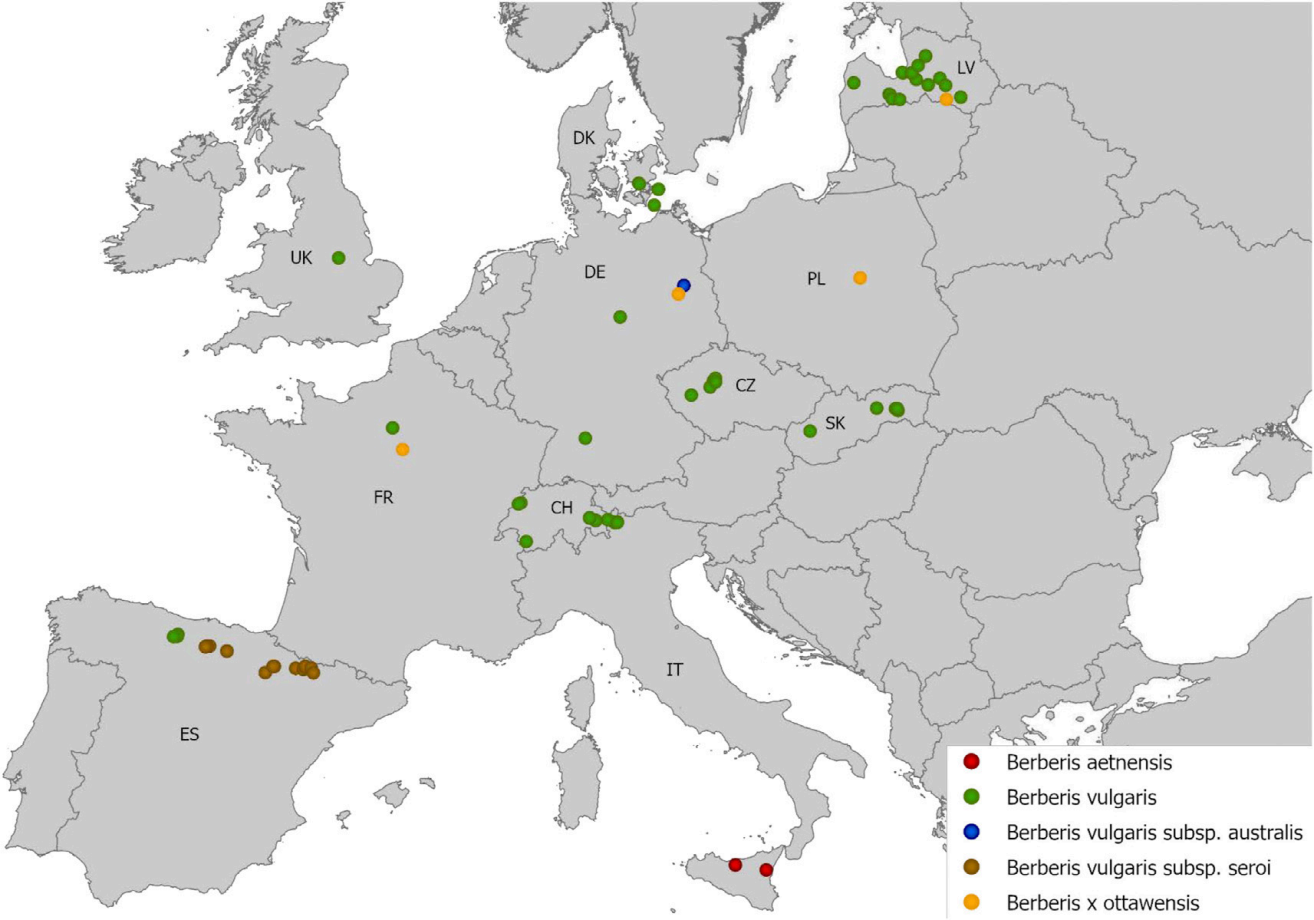
Infected barberry leaves were collected from five barberry species at multiple sites in 11 European countries, i.e., CZ: Czech Republic, CH: Switzerland, DE: Germany, DK: Denmark, ES: Spain, FR: France, IT: Italy, LV: Latvia, PL: Poland, SK: Slovakia, UK: United Kingdom (see Supplementary Table S1 for detailed survey information).

From 2018 to 2020, leaves bearing aecia of five barberry species, i.e., *B. vulgaris*, *B. aetnensis*, *B. vulgaris* subsp*. seroi*, *B. vulgaris* subsp*. australis,* and *B.* x *ottawensis* (an interspecific hybrid of *B. vulgaris* and *B. thunbergii*) were collected in 11 European countries (Figure 1). A total of 165 barberry samples, containing several rust infected leaves, were sampled from multiple sites with an elevation ranging from 1 to 2000 m above sea level (masl) and a distance to cereals and grass fields of approximately 0–20.000 m (Supplementary Table S1). Identification of barberry species was verified based on particular morphological characteristics, e.g., color of 1 year old stems, color of matured fruits, and type and morphology of racemes and leaves, according to Ahrendt (1961) and López González (1986). The infected leaves contained multiple aecial clusters varying in the number of aecial cups. Leaves were placed in glycine or paper bags to promote rapid drying and to avoid leaf curling. Infected barberry leaves were selected based on the non-degraded appearance of aecial clusters and were used for DNA sequencing and to investigate host specificities on cereal crops. Aecia of poor quality were only used for DNA sequencing.

### DNA sequencing and phylogenetic analysis of aecial clusters

Genomic DNA was extracted from dried single aecial clusters detached from barberry leaves. Single clusters were individually transferred to a 2 ml eppendorf tube containing approximately 20 mg acid washed sand and two steel balls (5 mm in diameter). Samples were pulverized in a 2010 Geno/Grinder (SPEX samplePrep), at 1,500 strokes per minute for 3 s × 20 s. Samples were extracted with the sbeadx plant kit (LGC genomics) using a KingFisher Magnetic Particle Processor for 24 samples (Thermo Scientific). DNA was extracted according to manufacturer’s instructions using 300 µl lysis buffer and 50 µl of the lysate for extraction. DNA was eluted in 50 µl elution buffer.

The PCR amplifications and sequencing of the elongation factor gene (EF1-α) region were conducted using the primers EFbasidR (Van Der Merwe et al., 2007) and EF1-α (Berlin et al., 2013a). These primers amplify a product of 650–680 bp of the EF1-α gene covering part of exon 2 and 5, and the full length of intron 2, exon 3, intron 3, exon 4, and intron 4, which amplifies from position 669 to 1,326 in the original EF1-α gene cloned from *P. graminis* (Schillberg et al., 1995). The PCR products waere amplified in a total reaction volume of 25 µl containing 1x GoTaq Flexi Buffer (5X, Promega, Germany), 1.5 mM MgCl_2_, 0.1 mM of each dNTP, 1 µM of each primer, 1 U GoTaq Flexi DNA polymerase (5 U/µl, Promega) and 1 µl of undiluted DNA. PCR conditions were 95°C for 3 min and 10 cycles at 95°C for 30 s, annealing at 60°C for 30 s, and 72°C for 2 min with 0.5°C decrease in annealing temperature per cycle followed by 30 cycles of 95°C for 30 s, annealing at 55°C for 30 s, and 72°C for 2 min and a final extension at 72°C for 2 min. PCR products were analyzed in an agarose gel electrophoresis assay and those which were successfully amplified were purified and bidirectionally sequenced with the same primers as for the PCR analysis using Sanger sequencing at Macrogen Europe BV, Amsterdam, Holland.

Forward and reverse sequences were assembled for each individual aecial sample using the QIAGEN CLC Genomics Workbench v. 20.0.4 (https://digitalinsights.qiagen.com) and apparent misalignments manually examined. After trimming, all sequences were aligned and a consensus sequence generated for each aecial sample. The Basic Local Alignment Search Tool (BLASTN) were used to find similarities between sequences in GenBank, NCBI (Altschul et al., 1997). Rust species identity was determined by the maximum identity observed compared to sequences available in GenBank. A maximum-likelihood tree was generated using the Neighbor Joining construction method with bootstrap values generated from 1,000 replicates. The General Time Reversible (GTR) nucleotide substitution model was used according to the model testing tool implemented in the QIAGEN CLC Genomics Workbench, which allow to identify the most appropriate substitution model to be used for maximum likelihood phylogeny tree construction (Yang, 1994). The resulting phylogenetic tree was visualized using iTOL v. 6.5.2 (Letunic and Bork, 2021). Five reference EF1-α sequences obtained from NCBI in addition to 15 internal reference sequences from multiple *Puccinia* species infecting various cereal and grass hosts were included in the phylogenetic analysis (Supplementary Table S2).

### Aecial host specificities

The capability of aecia to infect cereals was investigated by pooled aecia inoculations on varieties of wheat (*Triticum aestivum* var. Morocco and Line E), rye (*Secale cereale* var. Prolific), barley (*Hordeum vulgaris* var. Hiproly), and oat (*Avena sativa* var. Marvellous). for samples collected in Spain and United Kingdom, and var. Line E, Cartago, and Palazzo for samples collected in Switzerland, respectively. All varieties are considered as “universal” susceptible varieties of *P. graminis* ff.spp. including *Pgt* (var. Morocco, Line E, and Hiproly), *Pgs* (var. Line E, Hiproly, and Prolific), and *Pga* (var. Marvelous) (Villegas et al., 2022). Additionally, wheat varieties Cartago and Morocco were also included as these are considered as “universal” susceptible varieties for the yellow/stripe wheat rust fungus, *P. striiformis* f.sp. *tritici* (Milus et al., 2015; Hovmøller et al., 2017).

Twenty seeds of each variety were grown in square plastic pots and when the seedlings were approximately 2–4 cm, 3 ml of maleic hydrazine solution (Antergon^®^, MH 180, Crompton Registrations Ltd., Birmingham, England) was added to slow plant growth and enhance spore production. Two pots of each cereal variety were randomized and placed below barberry leaves bearing aecia, which were fixed to a plastic stand, misted with water and incubated at 15°C for 24 h in darkness, 100% relative humidity (RH). Subsequently, seedlings were transferred to spore-proof greenhouse cabins and kept at 18°C day/14°C night with a photoperiod of 16 h of natural light and supplementary artificial light (200 μmol·s^−1^ m^−2^), RH 80%. Uredinia resembling the stem rust fungus were observed from 10 days after aeciospore exposure on all cereal varieties. When clearly separated pustules developed, leaf segments of approximately 1–3 cm were detached and rinsed with water to remove possible spores from additional lesions. Leaf segments bearing uredinia were then dried for 2 days in a desiccator at room temperature and stored until further use.

### DNA extraction and molecular genotyping of uredinial isolates

The procedures described in Patpour et al. (2022) were used for genomic DNA extraction of *P. graminis* lesions sampled from the wheat, barley and rye varieties used in the aecial host specificity test and for molecular genotyping using 19 SSR loci designed for *Pgt*. Since these markers do not amplify samples derived from oat, the latter were not considered in this study.

### Population genetic analyses of uredinial isolates

The “*poppr*” package version 2.9.3 implemented in the R environment (version 4.2.0) was used for genetic analyses of sexually-derived uredinial isolates from three populations, i.e., Spain, United Kingdom and Switzerland, and cereal hosts, i.e., wheat, barley, and rye (Kamvar et al., 2014; Kamvar et al., 2015). Population genetic analyses included number of observed multilocus genotypes (MLGs) showing genotypic richness, G/N number of MLGs divided by number of samples, Simpson’s index (lambda) measuring genetic diversity (Simpson, 1949), evenness distribution of genotype abundance (E_5_) (Grünwald et al., 2003), and Nei’s unbiased gene diversity (H_exp_) (Nei, 1978). Analyses of molecular variance (AMOVA) and subsequent significant tests were performed on clone corrected data to evaluate molecular differences among populations and host of origin (Excoffier et al., 1992). The fixation index (F_st_), measuring pairwise divergence between populations, the number of effective migrants {Nm = [(1/F_st_)-1]/4} and Nei’s genetic distance were assessed using GenAlEx 6.503 with 9,999 permutations and bootstraps (Wright, 1965; Nei, 1972; Peakall and Smouse, 2006; Peakall and Smouse, 2012). Pearson’s correlation coefficients (*ρ*) were calculated to measure the linear correlation between F_st_, Nm, and Nei’s genetic distance values.

The population structure of related sexually-derived uredinial isolates was analysed by Discriminant Analysis of Principal Components (DAPCs) using the “*adegenet*” package version 2.1.5 implemented in the R environment (Jombart, 2008; Jombart et al., 2010). The DAPC, which transforms the MLG genetic data using Principal Component Analysis (PCA) and Discriminant Analysis (DA) on the PCs retained, is a multivariate non-parametric method without any predetermined population genetic model with respects to Hardy-Weinberg equilibrium or linkage disequilibrium (Jombart et al., 2010). The *find.clusters* function, without any predetermined information regarding the expected number of genetic groups, was used to identify the optimal number of clusters (K). This is normally associated with the lowest Bayesian Information Criterion (BIC) K value, which is frequently indicated by an elbow in the BIC curve (Jombart et al., 2010). As the number of PCs retained in the DAPC analysis has a strong impact in the final clustering, a cross-validation function (*xvalDapc*) with 1,000 replicates was implemented to find the optimal number of PCs to be retained, which was associated with the lowest root mean square error. Subsequently, a scatterplot with the first and second linear discriminants and the resultant clusters was generated. Finally, membership probabilities of isolates associated to a specific MLG and to a resultant cluster were calculated.

## Results

### Sequencing analysis of aecial clusters

**FIGURE 2:**
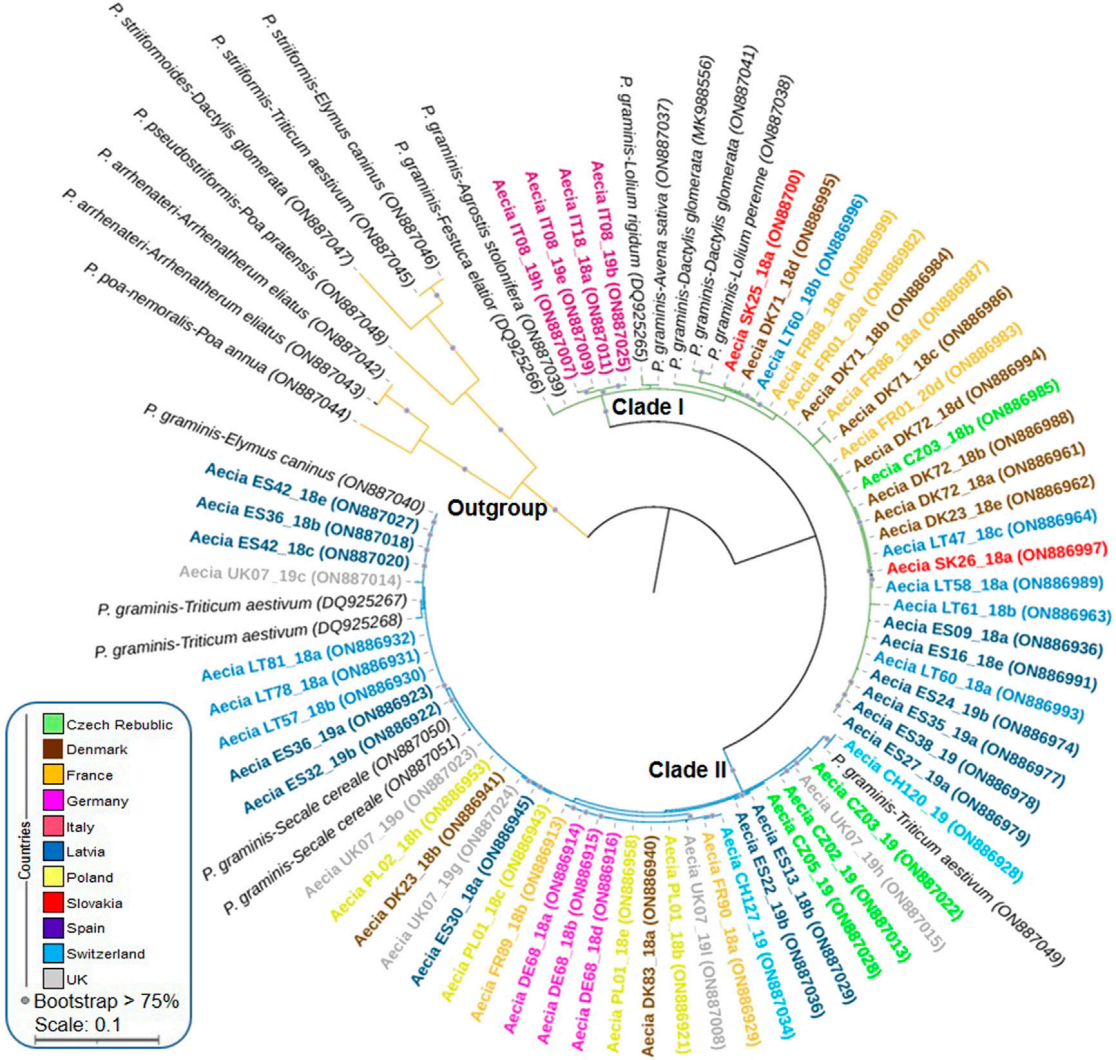
Phylogenetic analysis of the elongation factor 1-α (EF1-α) gene amplified for 61 aecial samples collected in 11 European countries and 20 *Puccinia graminis* and *P. striiformis* reference isolates collected from multiple cereals and grasses (accession numbers from NCBI). Sequences from *P. striiformis* and related species were included as an outgroup. Bootstrap values for 1,000 replicates are shown when >75%. Scale bar indicates nucleotide substitutions per site.

A total of 121 aecial samples from 11 EU countries were successfully sequenced and enabled species identification. All sequences had high identity values (from 98.0% to 99.4%) to *P. graminis* reference samples from NCBI collected from three different hosts, i.e., *T. aestivum, Dactylis glomerata,* and *Lolium rigidum* (Supplementary Table S3). For better visualization of sequence results, a clustering analysis using a subset of 61 aecial sequences representing all countries and hosts in addition to 20 *Puccinia* reference isolates collected from multiple cereal and grass hosts was performed. This resulted in the identification of two distinct clades (Figure 2; Supplementary Tables S2, S3). Clade I consisted of 29 aecial samples from seven countries with identity values corresponding to *P. graminis* samples collected from *D. glomerata* and *L. rigidum*, which grouped with *P. graminis* reference samples from *A. sativa* and other grasses, including *D. glomerata, L. rigidum, L. perenne, Festuca elatior* and *Agrostis stolonifera*. Some level of subgrouping was observed within Clade I, where aecial samples from Italy and most of the aecia from Spain, Denmark, and Latvia did not clearly group with any of the reference samples of *P. graminis*. Clade II comprised 32 aecial samples from nine countries with identity values corresponding to *P. graminis* samples collected from *T. aestivum*. These samples grouped with *P. graminis* reference samples from *T. aestivum, S. cereale,* and *Elymus caninus*, although no clear subgrouping was observed within this clade. None of the aecial samples grouped together with the *P. striiformis* reference isolate infecting wheat and related *Puccinia* species infecting multiple grasses, which clearly formed an outgroup (Figure 2).

### Aecial host specificity studies

Inoculation studies of aecial samples from three European countries, Spain, United Kingdom, and Switzerland, were successfully recovered and resulted in stem rust pustules on varieties of wheat, barley, rye, and oat, respectively, indicating the presence of multiple special forms of stem rust within the aecial samples investigated, e.g., *Pgt*, *Pgs,* and *Pga* (Table 1). A total of 533 single lesion isolates were recovered from these cereal hosts, and the amount varied across countries and cereal varieties. *P. striiformis* infecting cereals and grasses was not detected in any of the aecial samples.

**TABLE 1:** Number of single lesions of *Puccina graminis* derived from barberry leaves bearing aecia sampled in three European countries and recovered on multiple varieties of four major cereal crops (na, not assessed).

	Cereal crops						
Population	Wheat			Barley	Rye	Oat	
	Line E	Morocco	Cartago	Hiproly	Palazzo	Marvelous	Total
Spain	206	42	na	89	24	51	412
United Kingdom	19	4	na	29	7	0	59
Switzerland	1	na	19	na	42	na	62

### Genetic diversity and variance of sexual populations

Prior analysis, the suitability of the 19 SSR markers was assessed by generating a genotype accumulation curve (Supplementary Figure S1). This showed that the SSR loci applied in the present study were sufficient to discriminate between individuals in the dataset. Population genetic analyses was performed on a subset of 192 single stem rust isolates randomly selected according to host (wheat, rye, and barley) as well as geographical (Spain, United Kingdom, Switzerland) origin, which revealed 135 MLGs (Table 2; Supplementary Table S4). The allelic genotypes of each of the 192 isolates and their host of origin and corresponding MLG are provided in Supplementary Table S4. The highest number of MLGs was detected in the Spanish population followed by United Kingdom and Switzerland. Four MLGs (21, 27, 33, and 35) detected in the Spanish population were resampled from two host species, i.e., wheat and barley. High genotypic richness (0.53–0.93), genotypic diversity (0.93–0.99), evenness of genotype abundance (0.63–0.97), and gene diversity (0.64–0.81) were observed among the three sexual populations (Table 2).

**TABLE 2:** Genetic diversity parameters of sexually-derived *Puccinia graminis* isolates from three European populations at 19 microsatellite loci.

Population	N^b^	MLGs^c^	Genotypic richness^d^	Genotypic diversity^e^	Genotype abundance^f^	Gene diversity^g^
Spain	124	93	0.75	0.99	0.78	0.64
United Kingdom	53	28	0.53	0.96	0.63	0.73
Switzerland	15	14	0.93	0.93	0.97	0.81
Total^a^	192	135	0.70	0.99	0.69	0.74

a Indices calculated for pooled populations.

b Number of genotyped samples.

c Number of multilocus genotypes.

d Number of MLGs divided by number of genotyped samples.

e Simpson’s genotypic diversity index.

f Evenness of genotype abundance index (E_5_).

g Nei’s unbiased gene diversity (H_exp_).

The AMOVA results revealed significant variations by the two hierarchies analyzed (population and host) (Table 3). The highest variation was observed within samples for the two hierarchies (67%–72%) whereas genetic differences among samples within populations and hosts accounted for 19% and 27%, respectively. Variation among hosts accounted for only 1%. Variation among populations from the three countries explained 14% of the total variance. Subsequent individual AMOVA pairwise comparisons between populations revealed that the source of variation between Spain and United Kingdom increased up to 16%, decreased to 13% for Spain and Switzerland and significantly decreased to 2% for United Kingdom and Switzerland. The other two sources of variation (among samples within populations and within samples) resulted in similar values as those in Table 3 (Supplementary Table S5).

**TABLE 3:** Analysis of molecular variance (AMOVA) of *Puccinia graminis* isolates for two individual hierarchies (population and host) based on clone-corrected data.

Source of variation	Df^a^	Sum of squares	Mean of squares	Estimated variance	Variation (%)	*p-*value
Population						
Among populations	2	301.64	150.82	2.11	14	<0.001
Among samples within populations	132	2,153.95	16.32	2.95	19	<0.001
Within samples	135	1,406.04	10.42	10.42	67	<0.001
Host						
Among hosts	2	62.19	31.10	0.15	1	0.004
Among samples within hosts	136	2,466.94	18.14	3.89	27	<0.001
Within samples	139	1,439.32	10.36	10.36	72	<0.001

a Degrees of freedom.

*p*-values based on 1999 permutations.

### Structure and spatial differentiation of sexual populations

**FIGURE 3:**
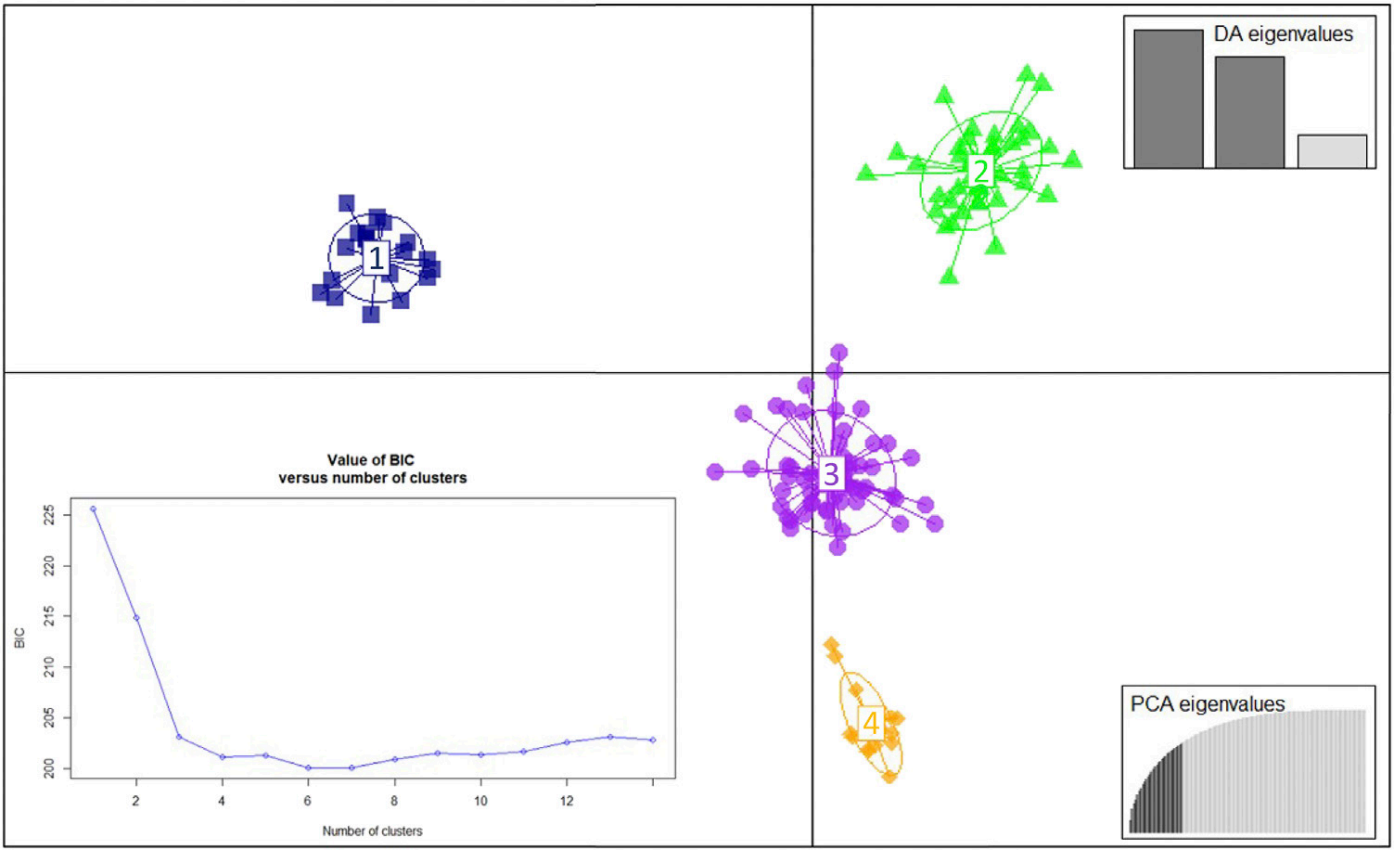
Discriminant analysis of principal components (DAPC) for the 135 multilocus genotypes (MLGs) detected among the 192 *Puccinia graminis* isolates. The Bayesian Information Criterion (BIC) supported four distinct genetic groups (bottom-left inset). The axes represent the first two linear discriminants. Each colored ellipsis represents distinct genetic clusters and symbols individual isolates. associated with unique MLGs. Eigenvalues indicate the amount of genetic information retained by the PCA (bottom-right inset) and the discriminant function (DA, top-right inset).

**FIGURE 4:**
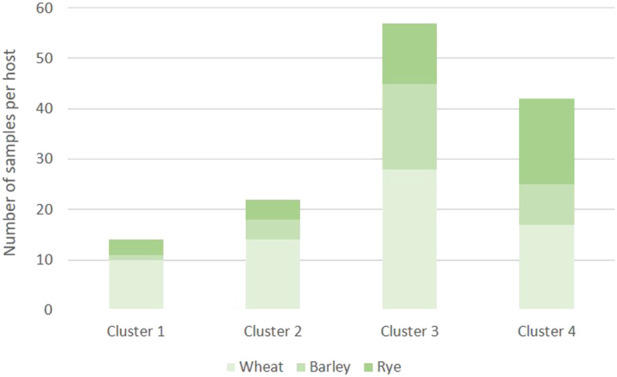
Number of *Puccinia graminis* samples originated from wheat, barley and rye and represented on each of the four clusters identified in the DAPC analysis.

To infer in the genetic structure of the three populations, a DAPC analysis was conducted using the 135 MLGs detected in this study. The DAPC analysis revealed four genetic clusters based on 20 PCs retained and three discriminant eigenvalues (Figure 2; Supplementary Figure S2). The optimal number of clusters corresponded with the lowest value of the elbow observed in the BIC curve, K = 4 (Figure 3). The 135 MLGs were clustered based on membership probabilities larger than 0.999 (Supplementary Table S6). Cluster 1, 2, and 3 consisted of 14, 22, and 57 isolates from the Spanish population, respectively. Each of these clusters corresponded with isolates derived from aecial samples collected in locally distinct barberry areas (Supplementary Figure S2; Supplementary Table S6). Cluster 4 consisted of 42 isolates from United Kingdom and Switzerland. Subsequent DAPC clustering at either K = 5 did not result in further subgrouping of CL4 (data not shown). No subgrouping was observed based on host of origin where samples originated from all three hosts (wheat, barley, and rye) were represented on each of the four clusters (Figure 4).

Pairwise comparisons for genetic differentiation between the genetic clusters (CL) detected by the DAPC analysis in addition to the original populations from Spain, United Kingdom, and Switzerland based on F_st_ values, Nei’s genetic distances and number of effective migrants (Nm) are shown in Table 4. Fixation indexes for the four genetic clusters detected in the DAPC analysis resulted in significant values ranging from 0.084 to 0.275, from 0.468 to 1.042 for the Nei’s genetic distance and from 0.775 to 2.875 for the Nm. This indicated a moderate to high genetic differentiation among CL1, CL2, and CL3 from isolates from Spain in addition to these three clusters compared to CL4, consisting of isolates form United Kingdom and Switzerland. Fixation indexes for the original population from Spain vs. United Kingdom and Switzerland resulted on significant values ranging from 0.084 to 0.125, from 0.446 to 0.708 for the Nei’s genetic distance and from 1.747 to 2.742 for the Nm. This implied a moderate to high genetic differentiation between the population from Spain compared to United Kingdom and Switzerland. In contrast, lower values of F_st_ and Nei’s genetic distance and higher values of Nm were observed for United Kingdom vs. Switzerland, suggesting a low genetic differentiation between these two populations. The correlation coefficient between the F_st_ and the Nei’s genetic distance values for the genetic clusters detected by DAPC analyses and the original populations from Spain, United Kingdom, and Switzerland ranged from 0.70 to 0.99, respectively, implying a high correlation between these two genetic differentiation parameters. Additionally, the number of effective migrants was inversely correlated to F_st_ (*ρ* = −0.94 and *ρ* = −0.97) and Nei’s genetic distance values (*ρ* = −0.80, *ρ* = −0.97).

**TABLE 4:** Pairwise genetic differentiation between the genetic clusters detected by DAPC analysis in addition to the original populations from Spain, United Kingdom, and Switzerland measured by the fixation index based on Wright’s F statistics (F_st_), Nei´s genetic distance and the number of effective migrants (Nm). Clone-corrected data confirmed the results.

	Fst	Nei´s genetic distance	Nm
Genetic clusters (CL)			
CL1 (Spain) vs. CL2 (Spain)	0,275*	0,680	0,775
CL1 (Spain) vs. CL3 (Spain)	0,084*	0,491	2,875
CL2 (Spain) vs. CL3 (Spain)	0,157*	0,468	1,433
CL1 (Spain) vs. CL4 (UK/Switzerland)	0,189*	0,818	1,115
CL2 (Spain) vs. CL4 (UK/Switzerland)	0,216*	1,042	0,882
CL3 (Spain) vs. CL4 (UK/Switzerland)	0,094*	0,564	2,430
Original populations			
Spain vs. United Kingdom	0,125*	0,708	1,747
Spain vs. Switzerland	0,084*	0,446	2,742
United Kingdom vs. Switzerland	0,051	0,220	4,696

Significant at *p* < 0.001.

## Discussion

Over the last decade, increasing reports of stem rust across Europe have raised concerns about the potential role of *Berberis* spp. in the epidemiology of stem rust infecting major cereal crops (Saunders et al., 2019; Patpour et al., 2022). Here, we identified the *P. graminis* species that infect the alternate host, barberry species, across Europe. Then, we evaluated which stem rust species undergo sexual reproduction and infect major cereal crops and investigated the genetic diversity and structure of sexually-derived populations infecting cereal hosts.

### Barberry species as source of rust inoculum

*Berberis* is a widely distributed genus with approximately 500 species described globally (Ahrendt, 1961). The current and widespread prevalence of *Berberis* spp., across Europe, mainly due to the repeal of eradication laws and the reintroduction for environmental purposes, emphasize the need to monitor the risks of spread of rust fungi from these to cereal crops (Berlin et al., 2013b; Saunders et al., 2019). By a systematic sampling of rust-infected barberry leaves across Europe, we were able to confirm the presence of multiple barberry species harboring aecial infections at multiple locations and under different ecological conditions. For instance, infected barberry plants were found in close proximity to cereal and grass crops at multiple locations across Europe, which may have a strong influence on the epidemiology of cereal rust fungi (Patpour et al., 2022). The barberry species identified included the widely distributed common barberry and three indigenous barberry species present in Spain and Italy. Additionally, aecial infections were sampled from *B.* x *ottawensis*, an interspecific hybrid of the susceptible common barberry and the resistant Japanese barberry*, B. thunbergii*. This highlights the importance of monitoring naturally occurring interspecific hybrids, which have previously been overlooked and may play a crucial role in the epidemiology of cereal rust fungi (Bartaula et al., 2018). Additionally, the presence of resistant barberry species such as *B. thunbergii,* either naturalized or as ornamentals, emphasizes the need to reconsider the potential risk that these may have in creating novel susceptible hybrids, which may have a profound effect in the epidemiology of cereal rust fungi.

### Identification of *Puccinia* species infecting barberry

In the present study, the EF1-α gene sequence was selected due to its suitability to distinguish closely related species within the *Puccinia* genus due to the presence of highly variable introns (Van Der Merwe et al., 2008). Sequencing analysis confirmed the presence of two main clades including multiple special forms of *P. graminis* infecting *Berberis* spp. across Europe. Clade I included *P. graminis* f.sp. *avenae*, which may infect oat and multiple grasses such as ryegrass (*L. perenne*) and orchard grass (*D. glomerata*). Clade II comprised *P. graminis* ff.spp. *tritici* and *secalis* infecting wheat and rye, respectively, in addition to *E. caninus* (aka bearded wheatgrass or bearded wild rye) (Stace, 2005). This is in line with previous studies using the EF1-α and the internal transcribed spacer (ITS) region where ff.spp. infecting wheat and rye were closely related whereas those infecting oat, ryegrass, and orchard grass clearly formed a separated group (Zambino and Szabo, 1993; Berlin et al., 2013a; Lewis et al., 2018). Certain level of subgrouping was observed within Clade I, which probably indicates the presence of additional special forms and/or subspecies infecting related wild grasses as stem rust can infect more than 300 grass species belonging to multiple genera (Anikster, 1984). Although multiple ff.spp. of *P. graminis* may coexist within the same area, hybridization between these appeared to be rather limited and restricted to occur at some degree between ff.spp. genetically related (Johnson, 1949). For example, sexual crosses between *Pgt* and *Pgs* resulted in the generation of viable progeny under controlled conditions. In contrast, crosses between *Pgt* and *Pga* rarely occur and lack the generation of aecial structures probably due to the involvement of reproductive barriers between non-genetically related ff.spp. (Johnson, 1949).

Additionally, *in vivo* experiments using aecial samples resulted in successful stem rust infections in susceptible varieties of wheat, barley, rye, and oat, indicating the presence of multiple ff.spp. of *P. graminis* infecting *Berberis* spp. in Europe. Stem rust lesions on wheat and rye indicated the presence of ff.spp. *tritici* and *secalis*, respectively. The same may be applied to the stem rust lesions obtained from barley, which could be a result of infections by either *Pgt* or *Pgs* or alternatively from crosses between these two ff.spp. (Anikster, 1984). In this study, four MLGs were resampled from wheat and barley, which further indicated that these were either derived from aecia of *Pgt* or *Pgs*. Stem rust lesions observed on oat indicated the presence of *Pga*. Although it could also be that the resulting stem rust infections were a result of different special forms and/or subspecies infecting other grass hosts as *P. graminis* is able to infect multiple grass species (Anikster, 1984). Accessory hosts such as wild cereals and grasses may play a crucial role in the epidemiology of stem rust as these could function as an important source of inoculum to maintain stem rust populations in the absence of cereal crops (Villegas et al., 2022). Host specificity analysis by cross-infection studies using a panel of different grasses and fungal strains, could help to obtain novel insights into host specialization and to evaluate the potential risk of stem rust spreading between different cereal and grass hosts.

Equally important as stem rust, the yellow rust fungus infecting wheat may undergo sexual reproduction on members of the *Berberidacea* family (Jin et al., 2010; Mehmood et al., 2020). The sexual cycle of *P. striiformis* has only been reported to occur in China at low frequencies, where barberry is ubiquitous and frequently growing alongside wheat crops (Zhao et al., 2013; Wang et al., 2016). Although *P. striiformis* infecting cereals and grasses was not detected in the present study, the increased prevalence of barberry spp. across Europe and the reported susceptibility under controlled conditions of multiple barberry spp. to *P. striiformis* strains widely present in Europe, stress the importance of continue searching in areas where conductive conditions for completion of the sexual cycle may occur (Rodriguez-Algaba et al., 2017, 2020, 2021).

### Genetic diversity and spatial differentiation

The application of neutral co-dominant molecular markers have proven very useful to infer in the genetic variability and structure among cereal rust populations (e.g., Berlin et al., 2012; Hovmøller et al., 2016; Kolmer et al., 2019). Here, the application of a set of SSR markers, previously applied on *Pgt* and some of them also proven useful on *Pgs*, performed equally well for samples derived from wheat, rye, and barley (Jin et al., 2009; Zhong et al., 2009; Berlin et al., 2012; Stoxen, 2012). Molecular genotyping applied on the progeny derived from three sexual populations of *P. graminis* confirmed that high genetic diversity was generated after sexual reproduction on the alternate host. This was exemplified by high number of MLGs and genetic diversity indexes. Sexual reproduction favors gene reshuffling leading to the generation of novel gene combinations with the potential of causing detrimental effects for cereal crop production (Roelfs and Groth, 1980; Jin, 2011). Indeed, high genetic variability and races carrying unusual virulence combinations associated with the sexual cycle have been reported using *P. graminis* samples collected from wheat, rye, and oat (Berlin et al., 2013b; Berlin et al., 2014; Olivera Firpo et al., 2017; Olivera et al., 2019). More recently, high genetic diversity with respect to virulence and microsatellite markers were detected in samples collected from wheat, rye, and creeping wild rye (*Elymus repens*) in Spain and from wheat and barley in Sweden in areas where the alternate host is locally present (Patpour et al., 2022).

Analysis of molecular variance of the sexual populations from Spain, United Kingdom and Switzerland revealed that the highest molecular variance was observed within and among samples, which is in line with the high genetic diversity observed based on the number of MLGs and diversity parameters. Genetic variation among populations explained 14% of the total variance, which significantly decreased to 2% in a pairwise comparison between sexual populations from United Kingdom and Switzerland. Pairwise comparisons for genetic differentiation between populations from United Kingdom and Switzerland based on F_st_ values, Nei’s genetic distance and Nm revealed a moderate to low genetic differentiation and high gene flow between these two populations (Wright, 1965; Nei, 1972; Sharma-Poudyal et al., 2020). In contrast, the comparison between the Spanish population and the sexual populations from United Kingdom and Switzerland suggested a significantly higher genetic differentiation and lower genetic exchange. The DAPC, a multivariate analysis without any predetermination with respects to Hardy-Weinberg equilibrium or linkage disequilibrium (Jombart et al., 2010), provided additional clues about the genetic structure of the sexual populations. Four distinct clusters were identified, where three of them belonged to *P. graminis* samples derived from three locally separated locations in Spain. These three subpopulations were characterized by high F_st_ and Nei’s values, and relatively low number of migrants. This suggested that each of the Spanish subpopulations were formed as a result of fungal infections generated on barberry plants infected with local *P. graminis* populations with low genetic exchange. A fourth cluster was comprised of isolates derived from United Kingdom and Switzerland. This is consistent with the low genetic differentiation observed between these two populations based on results from AMOVA, F_st_, Nei’s genetic distance and Nm values. Indeed, gene flow due to aerial dispersal of fungal spores over long distances is a common phenomenon previously reported for rust fungi (Nagarajan and Singh, 1990; Brown and Hovmøller, 2002). An additional explanation of the low genetic differentiation observed between the populations from United Kingdom and Switzerland could be that both sexual populations were originated by common parental ancestors which underwent sexual reproduction on distant barberry areas. Lastly, the DAPC analysis revealed no sub-clustering based on host of origin of the sexually-derived isolates infecting wheat, rye, and barley. As previously indicated, the lack of differentiation between ff.spp. infecting wheat, rye and barley could be due to genetic similarities between these ff.spp. and probably the existence of some level of overlap in host range.

This study demonstrated that a range of barberry species across multiple sites in Europe were functionally active as sexual host for stem rust infecting cereals. This underlines the need to reinitiate rust resistance breeding strategies to enhance cereal crop defenses in case the sexual host becomes a significant part of the epidemiology of stem rust in Europe. Future screen of cereal germplasm commonly grown in Europe using sexual-derived populations of *P. graminis* will allow to evaluate the current level of stem rust susceptibility and assess which resistance genes are more likely to evolve towards new virulences and hence put at risk the durability of specific resistance genes. This will help to select resistance genes and combinations thereof, which are expected to be more durable, and develop more sustainable disease management and resistance deployment strategies.

## Data Availability

The original contributions presented in the study are included in the article/Supplementary Material. The EF1-α sequence data reported in the present study were deposited in GenBank repository (https://www.ncbi.nlm.nih.gov/genbank/) under the accession numbers listed in Supplementary Tables S2,S3, i.e., ON886913-ON887051.
