# Supplementary material for: Stem rust on barberry species in Europe: Host specificities and genetic diversity
Source: Front Genet. 2022 Sep 27;13:988031. doi: 10.3389/fgene.2022.988031 (PMC9554944; doi:10.3389/fgene.2022.988031)
Supplement: Supplementary file 1 [file DataSheet1.PDF]

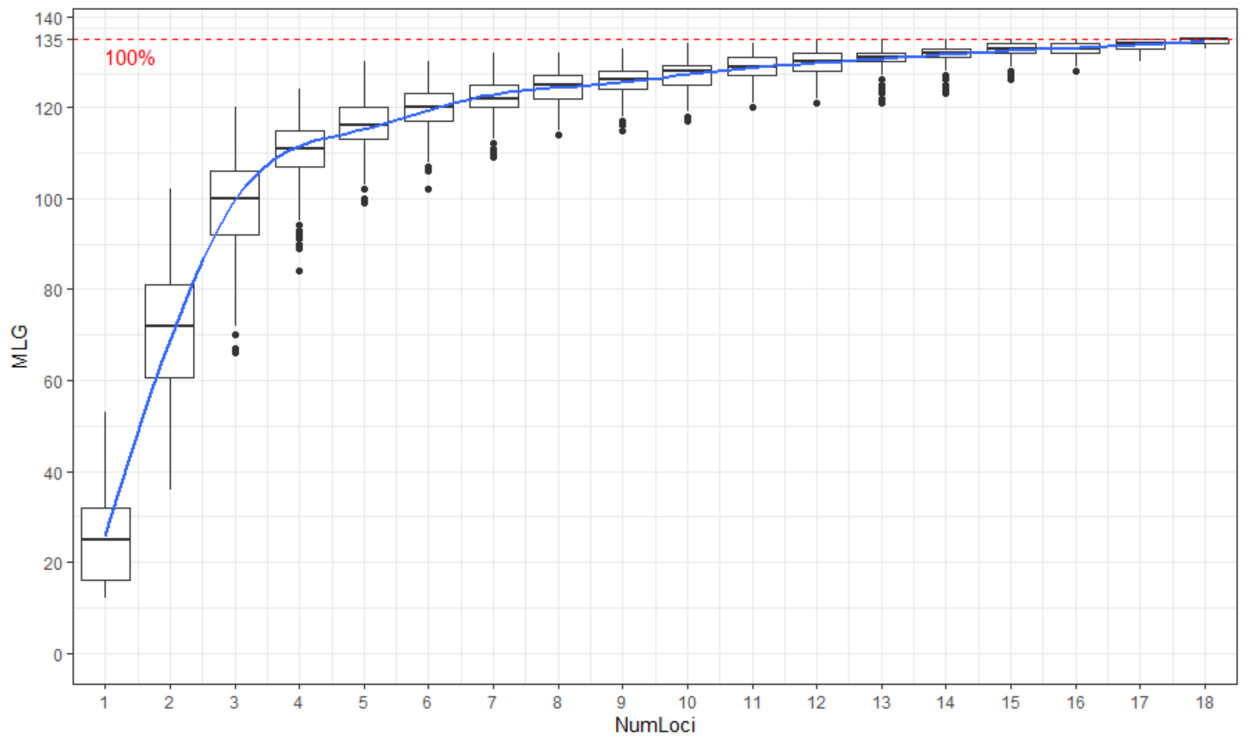


**Supplementary Figure 1**. Genotype accumulation curve with the number of loci (n-1, maximum number of loci) and the 135 discriminated MLGs.  The 19 SSR loci applied in the present study were sufficient to discriminate between *P. graminis* individuals in the dataset.

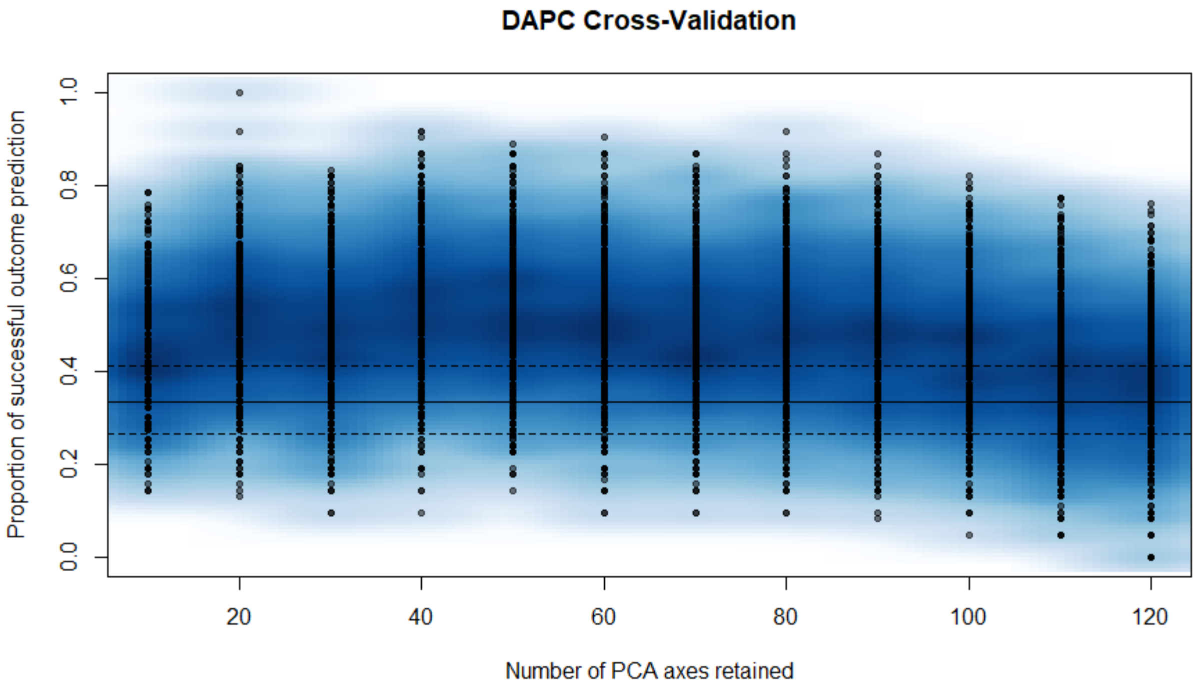


**Supplementary Figure 2**. Cross-validation of the discriminate analyses of principle components (DAPC). Each dot represents individual replicates, which supported 20 principal components (PCAs) to be retained in the analysis.

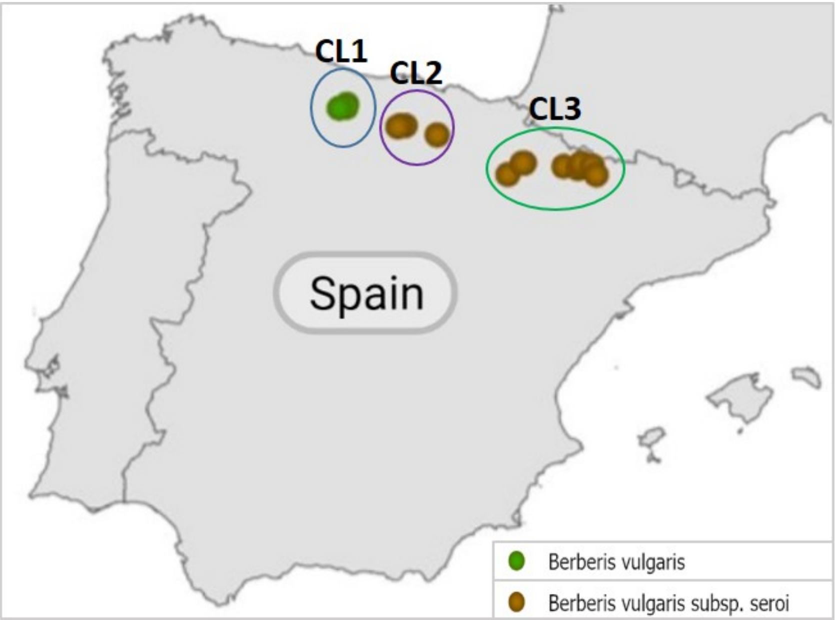


**Supplementary Figure 3.** Cluster 1, 2 and 3 were comprised of isolates collected from local and distinct barberry areas in Spain.
